# Phosphatidylcholine Cytidine Transferase α (CCTα) Affects LD Formation Through Fusion and Lipophagy in Bovine Mammary Epithelial Cells

**DOI:** 10.3390/ijms26052135

**Published:** 2025-02-27

**Authors:** Jingna Yang, Yuxin Fan, Fangyuan Kang, Yanbin Yang, Yueying Wang, Yang Liu, Liqiang Han

**Affiliations:** Key Laboratory of Animal Biochemistry and Nutrition, Ministry of Agriculture and Rural Affairs, College of Veterinary Medicine, Henan Agricultural University, Zhengzhou 450046, China; yangjingna2025@126.com (J.Y.); z764443374@163.com (Y.F.); fykang2025@163.com (F.K.); yangyb@henau.edu.cn (Y.Y.); wangyueying2008@126.com (Y.W.)

**Keywords:** lipid droplet, bovine mammary epithelial cell, phosphatidylcholine cytidine transferase α, fusion, lipophagy

## Abstract

Phosphatidylcholine cytidine transferase α (CCTα) is a key rate-limiting enzyme in the CDP–choline pathway, the primary pathway for phosphatidylcholine (PC) synthesis in mammals. This study investigated the role of CCTα in lipid droplet (LD) formation, phospholipid synthesis, LD fusion, and lipophagy in bovine mammary epithelial cells (BMECs) through CCTα gene knockout (CCT-KO) and overexpression (CCT-OE). CCTα mRNA expression was significantly increased in bovine mammary gland tissue after lactation. In BMECs, CCTα was transferred from the nucleus to the endoplasmic reticulum and localized on LD surfaces in the presence of linoleic acid. Compared with normal BMECs (NC), CCTα knockout (CCT-KO) cells had significantly greater LD diameters (1.53 μm vs. 1.68 μm, *p* < 0.05), lower proportions of small LDs (<1 µm; 11.39% vs. 5.42%), and higher proportions of large LDs (>3 µm; 0.67% vs. 2.88%). In contrast, CCTα overexpression (CCT-OE) decreased the diameter of LDs to 1.18 μm (*p* < 0.01), increased the proportion of small LDs to 35.48%, and decreased the proportion of large LDs to 0.24%. CCTα knockout significantly decreased the PC content and the ratio of PC to PE, whereas CCTα overexpression increased the PC content and the ratio of PC to phosphatidyl ethanolamine (PE) (*p* < 0.05). The lipidomics analysis indicated that PC synthesis was significantly influenced by CCTα gene expression. Live cell observations showed that CCTα knockout promoted the fusion of small LDs into large LDs. In cells with CCT α overexpression, the expression of the microtubule-associated protein 1 light chain 3 (LC3) protein and the number of lysosomes was elevated, and the lysosomal phagocytosis of LDs was observed through transmission electron microscopy, thus indicating that CCTα overexpression enhanced lipophagy. In conclusion, these results suggest that CCTα plays a role in regulating LD formation by influencing PC synthesis, LD fusion, and lipophagy in BMECs.

## 1. Introduction

Lipid droplets (LDs) are storage organelles central to lipid and energy homeostasis [1]. LD biogenesis is closely associated with cell homeostasis and metabolism [2]. LDs consist primarily of a neutral lipid core surrounded by a single layer of phospholipids and proteins [3]. The formation of intracellular LDs is closely associated with cellular needs, and the LD size and number vary according to LD composition [4]. The bovine mammary epithelial cells (BMECs) are the cellular basis for the mammary gland to exercise lactation functions and are functional cells for lipid droplet synthesis and secretion. The LDs in BMECs are the precursors for the synthesis of milk fat globules. They are transported to the apical plasma membrane and secreted into the milk through exocytosis, forming milk fat globules [5]. The PC content of fat globules is related to the size of milk fat globules, and there is an intrinsic correlation between the size of lipid droplets and the diameter of milk fat globules [6,7]. Therefore, LDs affect the content of phospholipids and proteins in milk fat globules and play crucial roles in the quality of milk and milk products, thus indirectly affecting neonatal development [7,8]. Therefore, studying the regulatory mechanism of LD formation in BMECs is important for maternal and infant health.

Studies in adipose [9] and liver [10] cells have shown that phospholipids regulate the formation of LDs. Phosphatidylcholine (PC), the most abundant phospholipid component in LDs, regulates the size and quantity of LDs in nematodes [11] and Drosophila [12]. The CDP–choline pathway produces most of the PC in mammalian cells [13], and phosphatidylcholine cytidine transferase α (CCTα) is an important rate-limiting enzyme in this process, which affects the synthesis rate of PC. In rat intestinal epithelial cells, CCTα silencing increases the LD size, but decreases the LD number and TG deposition [14]. The knockout of CCT in CaCO2 cells results in a 50% decrease in PC synthesis and enhances the storage of TG in large LDs [15]. The LD size and number depend on the balance between synthesis and degradation. Several pathways of LD formation and degradation exist, including LD fusion, intracellular lipolysis, and autophagy [4,16]. However, the mechanisms of CCTα in LD formation and regulation remain unclear.

Our previous studies in BMECs have shown that genetic effects on LD formation can be studied through knockout or overexpression [17]. Therefore, in this study, the key enzyme CCTα was knocked out or overexpressed, and its effects on LD formation and phospholipid synthesis in BMECs were observed. We also analyzed effects of CCTα expression on cellular LD fusion and lipophagy. Our findings confirmed that CCTα is an important factor affecting the formation of LDs in bovine mammary tissue.

## 2. Results

### 2.1. Expression Levels of CCTα in Bovine Mammary Gland Tissues

**Figure 1:**
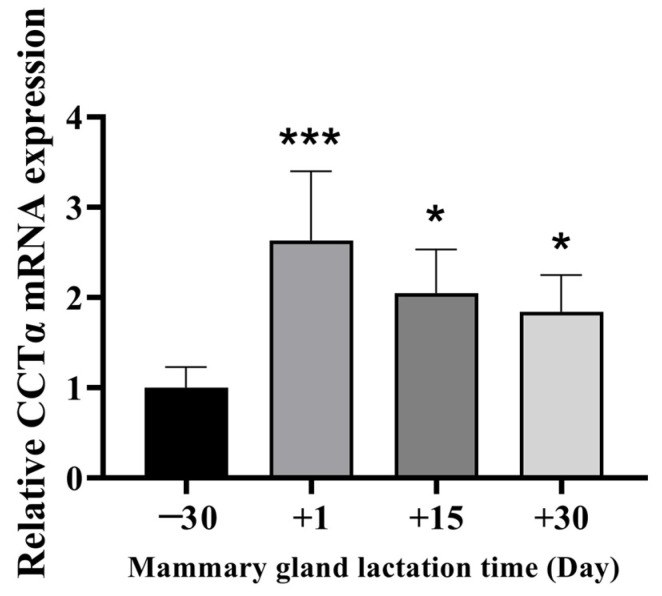
Expression of CCTα mRNA in bovine mammary glands at −30, 1, 15, and 30 d relative to parturition. All data are presented as mean ± SD; * *p* < 0.05; *** *p* < 0.001.

According to real-time quantification PCR (RT-qPCR), the expression of CCTα in mammary tissue on days 1, 15, and day 30 of lactation, compared with 30 days before lactation, significantly increased, by 3.0 times, 2.1 times, and 1.5 times, respectively (*p* < 0.05) (Figure 1). The expression level of CCTα in lactating mammary gland tissue was significantly higher than that in the dry period, thus suggesting that CCTα has a potential regulatory role in bovine mammary lactation.

### 2.2. CCTα Shuttles Between the Nucleus and Cytoplasm

**Figure 2:**
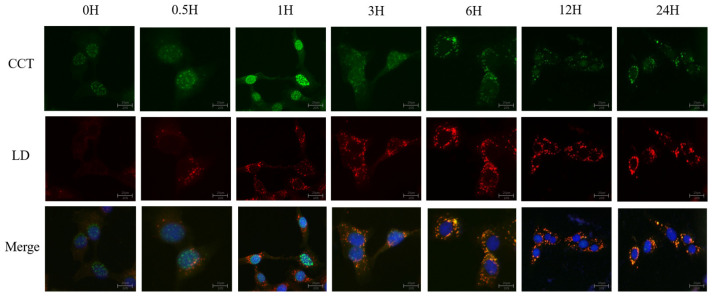
Co-localization of LDs and CCTα. LDs are in red, nuclei are stained blue, and CCTα protein is in green in BMECs.

To explore CCTα localization in BMECs, we conducted immunofluorescence staining on cells treated with 100 µM of linoleic acid (LA) (Figure 2). CCTα protein was stained with green fluorescence, LD is shown in red, and nuclei are shown in blue. at 0 h, the green fluorescence co-localized primarily with the blue fluorescence; therefore, CCTα was localized in the nucleus. At 6 h, 12 h, and 24 h, with the action of LA, lipid droplets were continuously produced, and the green fluorescence gradually transferred from the nucleus to the cytoplasm and co-localized with the red fluorescence, thus suggesting that CCTα was transferred from the nucleus to the cytoplasm in the presence of LA, and co-localized with LDs.

### 2.3. Construction of CCTα Knockout and Overexpression Cell Lines

To explore the effects of CCTα on LDs in BMECs, we constructed CCTα knockout (CCT-KO) and overexpression (CCT-OE) cell lines. The mRNA and protein expression of CCT was detected with WB and qPCR. In the CCT-KO cells, CCTα mRNA was significantly down-regulated, and the protein was undetectable, whereas the mRNA and protein levels were significantly up-regulated in CCT-OE cells (Supplementary Figure S1). Sequencing verification (Supplementary Figures S4 and S6) indicated that the CCTα knockout and overexpression cell lines were successfully established.

### 2.4. Effects of CCTα on LD Size and Number

**Figure 3:**
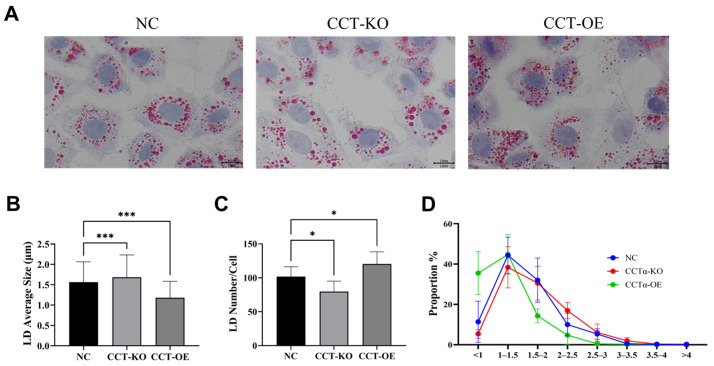
Effects of CCTα on LD size and number in BMECs. (**A**) Oil red O staining of LDs. LDs are shown in red, and nuclei are shown in purple-blue. (**B**) LD average size. (**C**) The average number of LDs around every cell. (**D**) Effects of CCTα on the proportions of LD sizes in BMECs. All data are presented as mean ± SD; * *p* < 0.05; *** *p* < 0.001.

To further observe the effects of CCTα knockout and overexpression on LD formation, we performed Oil Red O staining to analyze the LD size and number (Figure 3A). Compared with the average size of wild-type BMECs (NC; 1.56 μm), the CCTα knockout had a significantly greater LD size (1.68 μm), whereas the CCTα overexpression line had a smaller LD size (1.18 μm) (Figure 3B). Compared with the NC group, in the CCT-KO group, the LD number was significantly lower, at 79/cell (*p* < 0.05), whereas in the CCT-OE group, the LD number was higher, at 120/cell (Figure 3C). In the CCT-KO group, the proportion of large LDs (size > 3 μm) increased from 0.67% to 2.88%, and the proportion of small LDs (size < 1 μm) decreased from 11.39% to 5.42%, whereas in the CCT-OE group, the proportion of small LDs increased to 35.48%, and that of large LDs decreased to 0.24% (Figure 3D). These results indicated that CCTα knockout increased the size of LDs and the proportion of large LDs, whereas CCTα overexpression decreased the size of LDs and the proportion of large LDs.

### 2.5. Effects of CCTα on Phospholipid Synthesis

**Figure 4:**
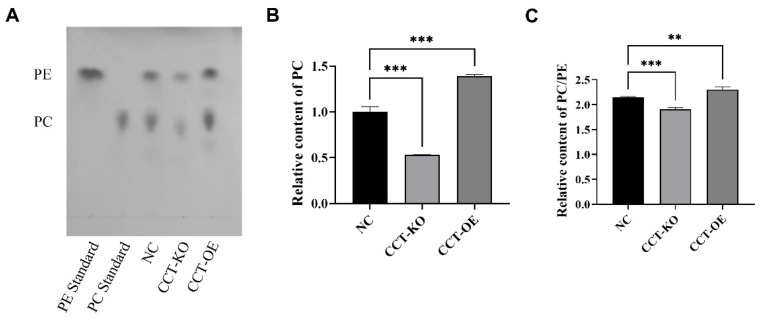
Analysis of phosphatidylcholine (PC) and phosphatidylethanolamine (PE) content. (**A**) Thin layer chromatography analysis of PC and PE. (**B**) Relative content of PC. (**C**) PC/PE ratio. All data are presented as mean ± SD; ** *p* < 0.01; *** *p* < 0.001.

PC and phosphatidyl ethanolamine (PE) are the main components of phospholipids on LD surfaces. The quantitative analysis of the PC and PE content showed that the CCTα knockout significantly decreased the PC content and PC/PE, whereas CCTα overexpression significantly increased the PC content and PC/PE (Figure 4). These results suggested that CCTα affects PC synthesis.

**Figure 5:**
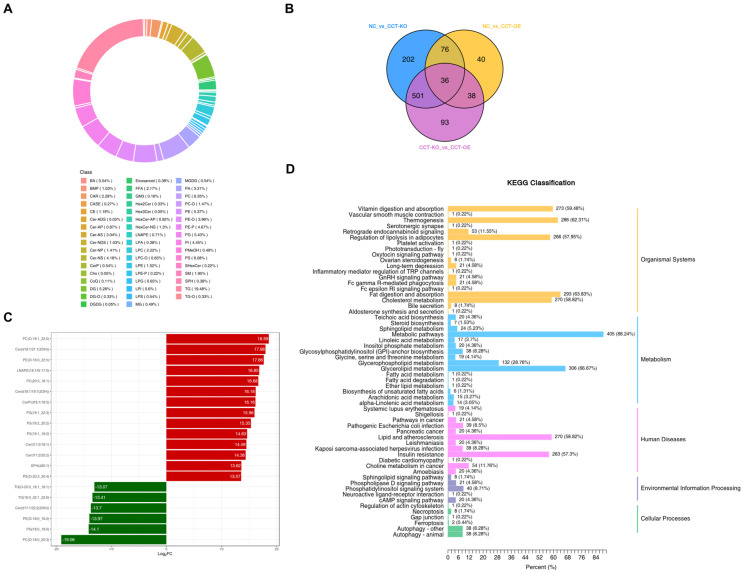
Effects of CCTα on cell lipid content and pathways. (**A**) Cell lipid classification. Each color represents a lipid subclass, and the color-blocked area represents the proportion of this category. (**B**) Amounts of differential lipids among groups. (**C**) Top 20 differential lipids. The abscissa is log_2_FC, and the ordinate is differential lipids. Red indicates up-regulation of lipid content, and green indicates down-regulation of lipid content. (**D**) KEGG classification map of differential lipids. The ordinate is the name of the KEGG metabolic pathway, and the abscissa is the number of differential lipids annotated to the pathway and its proportion with respect to the total annotated lipids.

To further investigate the effects of CCTα knockout and overexpression on phospholipids synthesis, we assessed differential phospholipids through lipidomics. A total of 1843 lipid compounds were identified (Table 1). Compared with the control group, CCTα overexpression increased the relative content of 140 lipid compounds and decreased the relative content of 50 lipid compounds. The CCTα knockout decreased the relative content of 48 lipid compounds and increased the relative content of 767 lipid compounds. PC (6.35%) and PE (5.37%) were the main glycerophospholipid components (Figure 5A). A total of 36 differential phospholipids were identified among the NC group, CCT-KO group, and CCT-OE group (Figure 5B).

We compared lipids between the knockout and overexpression groups and identified 597 down-regulated and 74 up-regulated lipids (Table 1). Moreover, among the top 20 lipids with the most significant differences, the highest and lowest differentiated lipid classes were PC (o-18:1 22:0) and PC (o-18:0 22:3) (Figure 5C). The KEGG analysis of differential lipids showed that they were mainly involved in fatty acid metabolism, lipid metabolism, and disease and autophagy related pathways (Figure 5D). Combined with the results in Figure 4, these findings indicated that the knockout and overexpression of CCTα significantly affect PC synthesis, as well as the content of other lipid compounds in cells.

### 2.6. CCTα Knockout Promotes LD Fusion and Generation of Large LDs

**Figure 6:**
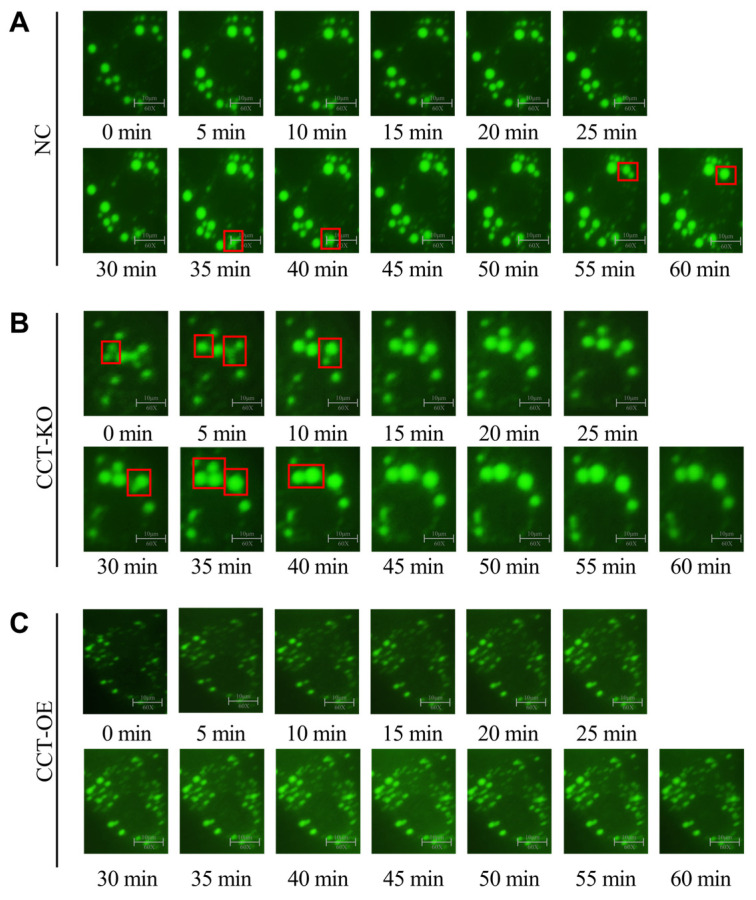
Effect of CCTα on LD fusion. (**A**) LD fusion in the NC group. (**B**) LD fusion in the CCT-KO group. (**C**) LD fusion in the CCT-OE group. Cells were stained with BODIPY493/503, and photographs were taken every 5 min for 60 min with a live cell workstation. Red box indicates a fusion of LD.

Two or more LDs can approach each other and fuse into a larger LD. CCTα-KO increased the proportion of large LDs and therefore might promote LD fusion. To validate this possibility, we used a live cell workstation and took photographs every 5 min to observe LD fusion under the visual field. Within 60 min, LD fusion occurred four times (at 0–5 min, 5–10 min, 30–35 min, and 35–40 min; Figure 6B) in the CCT-KO group, whereas in the normal group, LD fusion occurred twice (at 35–40 min and 55–60 min; Figure 6A), and the CCT-OE group did not show significant LD fusion in 60 min (Figure 6C). These results indicated that the CCTα knockout promoted the fusion of small LDs into large LDs, thereby increasing the size and proportion of large LDs.

### 2.7. CCTα Overexpression Enhances Lipophagy

**Figure 7:**
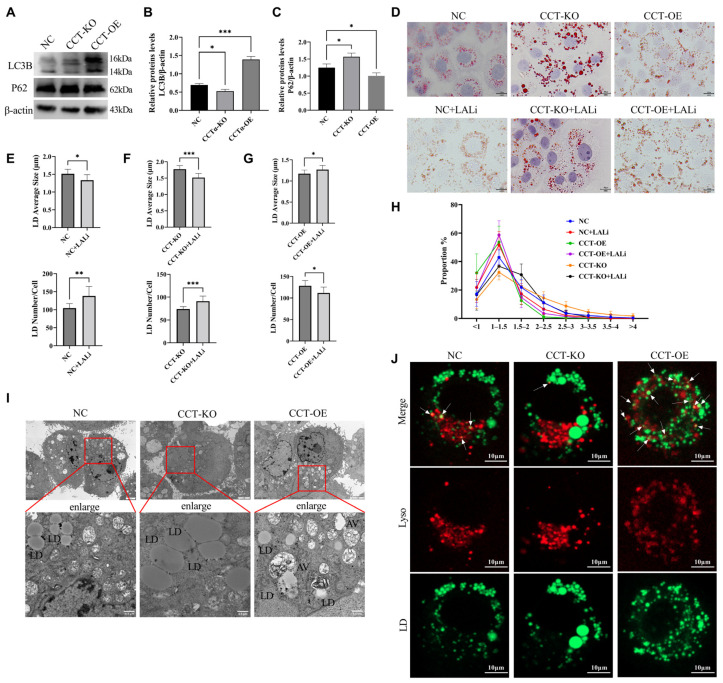
Effect of CCTα on cell lipophagy. (**A**–**C**) Protein expression of LC3 and p62. (**D**) Oil Red O staining of LDs. (**E**–**G**) Number and average size of LDs. (**H**) LD size proportions. (**I**) Transmission electron microscopy. LD: lipid droplet; AV: autophagy lysosome. The image on the bottom is a partial enlargement of the image on the top. (**J**) Lysosome staining. LDs are stained green with BODIPY493/503, and lysosomes are stained red with Lyso-tracker. The white arrow indicates LD and lysosome co-localization. All data are presented as mean ± SD; * *p* < 0.05; ** *p* < 0.01; *** *p* < 0.001.

Lipophagy is an important factor regulating LD size. The expression of the autophagy marker microtubule-associated protein 1 light chain 3 beta (LC3B) was significantly elevated in the CCT-OE cells (Figure 7A–C). To confirm the role of autophagy, we inhibited autophagy with lysosome inhibitors (LALi) and detected the effects on the LD size (Figure 7D). In the NC group, lysosome inhibition significantly decreased the LD diameter and increased the number of LDs, and the proportion of large LDs decreased, whereas the proportion of small LDs increased (Figure 7E). In the CCT-KO group, LDs showed the same changes (Figure 7F) as those in the NC group, whereas in the CCT-OE group, lysosome inhibition increased the LD diameter and decreased the LD number (Figure 7G), thus decreasing the proportion of small LDs and increasing the proportion of large LDs (Figure 7H). These results suggested that autophagy in CCT-OE affects the size of LDs.

LD and lysosome co-localization was observed through fluorescence staining. The number of lysosomes and the co-localization of LDs with lysosomes was elevated in the CCT-OE group (Figure 7J). Furthermore, we used transmission electron microscopy to observe the localization of LDs and autophagic lysosomes (Figure 7I). LDs and lysosomes were separated from each other in the control group and CCT-KO group, whereas in the CCT-OE group, the lysosomes clearly phagocytosed LDs, thus indicating lipophagy. These results suggested that CCTα overexpression increases the co-localization of lysosomes with LDs, thereby enhancing lipophagy.

## 3. Discussion

LDs are intracellular organelles that store neutral lipids. Fatty acid synthesizes LDs in the endoplasmic reticulum in mammary epithelial cells [18,19]. In the present study, we identified the mechanism of CCTα in LD formation in BMECs, which involves the CCT-PC pathways and specific metabolic processes (fusion or lipophagy) in the intracellular compartments. First, we verified the mRNA expression level of CCTα in mammary tissue and observed that CCTα was significantly higher during the lactation period than the dry period. In BMECs, CCTα underwent nucleoplasmic transfer, thereby influencing PC synthesis. The knockout of cellular CCTα decreased the PC/PE ratio, enhancing LD fusion and forming of larger LDs, thus promoting LD growth. In contrast, CCTα overexpression increased PC/PE and up-regulated LD degradation via lysosomal lipase, thereby decreasing the LD size.

The results of this study showed that the mRNA expression level of CCTα was significantly increased after lactation; CCTα is a key enzyme involved in the synthesis of PC, a major component of milk fat globules. During lactation, the mammary glands require a large amount of PC to produce milk fat, which is essential for the formation and stability of MFGs. To meet the increased demand for PC and lipids in milk, the expression of CCTα is up-regulated in lactating mammary tissue. This ensures there is an adequate supply of PC for MFG formation, which in turn contributes to the production of milk fat. Although studies have shown that CCTα affects intracellular lipid synthesis, the specific role of CCTα in the lipid composition of milk fat is still unknown. During lactation, dairy cows have a high metabolic demand for milk production, especially for the synthesis of milk fat and phospholipids. Therefore, the increased CCTα expression of post-lactation may be part of an adaptive response to metabolic demands.

CCTα is a nuclear enzyme with a nuclear localization signal in its amino terminal sequence [20]. After stimulation with a lipid activator [21], CCTα translocates to the cytoplasm, where it performs PC synthesis [22,23]. In the present study, we used LA as a lipid activator to construct the LD model. During the initial stage (0.5–1 h), CCTα was predominantly localized in the nucleus. After the LA treatment, CCTα began to be transported to the cytoplasm and finally localized on the surfaces of LDs, where it exerted regulatory effects on LDs. Similarly, Krahmer has found that CCTα is recruited to the phospholipid monolayer of LDs when fatty acids are added to cells to promote the synthesis of LDs [24]. In oleate-treated insect cells, LD binding to CCTα has been proposed to increase PC synthesis and consequently LD expansion [12,25]. The present results suggested that CCTα shuttles between the nucleus and cytoplasm upon a phosphatidylcholine demand in BMECs.

CCTα directly catalyzes the rate-limiting step in the biosynthesis of PC via the Kennedy pathway [26]. It catalyzes the conversion of CTP and phosphocholine to CDP–choline, which is subsequently used to synthesize PC. The synthesis of PC is essential for the formation of lipid droplets because PC is a key component of the lipid droplet monolayer [13]. CCTα increases the availability of PC, ensuring the proper composition and structural integrity of lipid droplets. In addition, PC plays a key role in the expansion and stability of the lipid droplet membrane, and the CCTα-mediated regulation of PC synthesis regulated the formation of larger lipid droplets [24]. The silencing of CCTα in 3T3-L1 cells decreases the number of LDs and increases the LD size [14]. In this study, the knockout of CCTα significantly increased the LD size, decreased the LD number, decreased the proportion of small LDs, and increased the proportion of large LDs, whereas the overexpression of CCTα elicited the opposite results. In CaCO_2_ cells, the knockout of CCTα has been found to result in fewer and larger LDs [15]. Studies in macrophages have shown that CCTα is recruited to the surfaces of inflated LDs, and the consumption of CCTα leads to formation of large LDs [24]. In rat intestinal epithelial cells, CCTα silencing increases the LD size but decreases the LD number [14]. These results suggest that CCTα plays a regulatory role in the LD size and number in BMECs.

LDs consist of neutral lipid nuclei, which are surrounded by a layer of phospholipids, of which PC and PE are the main constituents. Depending on cell type, PC is produced through three biosynthetic pathways: the CDP–choline pathway [24], PE methylation [9], and Lands pathway lysophosphatidylcholine acyltransferases (LPCATs) [27]. As the rate-limiting enzyme in the CDP–choline pathway, CCTα converts phosphocholine to CDP–choline; then, under catalysis by choline phosphotransferase, CDP–choline and diacylglycerol synthesize phosphatidylcholine in the endoplasmic reticulum [26]. In CaCO_2_ cells, the knockout of CCTα decreases PC synthesis by 50% [15]. In this study, the overexpression and knockout of CCTα affected PC synthesis through the CDP–choline pathway. The CCTα knockout decreased the PC content and PC/PE ratio, whereas CCTα overexpression increased the PC content and PC/PE ratio (Figure 4). Some evidence indicates that the synthesis pathways of SL, PL, and TG are interconnected and have a significant impact on the composition and size of LDs [28]. When the CDP–choline pathway affects the synthesis of PC, the relative contents of lipids in other PC-synthesizing pathways, such as PE, TG, diacylglycerol (DG), sphingomyelin (SM), and ceramide (Cer), also change, further influencing lipid droplets. Our research findings also demonstrate correlations among some differential lipids (Supplementary Figure S7).

CCTα supplies PC for LD expansion and stabilization in mammalian cells [26]. The silencing of CCTα in 3T3-L1 cells has been found to inhibit PC synthesis [14]. PC is the major surface lipid on LDs, and the expansion of the LD surface during LD growth requires a source of PC [19,29]. Studies have shown that the ratio between PC and PE determines the stability of LDs; decreasing the PC/PE ratio in the monolayer surrounding the intracellular LD has been predicted to promote LD fusion [30,31]. Bat-Chen Cohen has suggested that small intracellular LDs have elevated PC/PE ratios after palmitic acid treatment [32]. Similar changes in the PC/PE ratio and droplet size have been observed in differentiating preadipocytes [9]. In 3T3-L1 cells, PC synthesis occurs through a synergistic pathway involving increased phosphatidylserine synthesis and decarboxylation, followed by PE methylation to form PC [9]. PE can be methylated into PC by PEMT; PE and PC can be converted into PS by the PSS enzyme; and PE and PC can be converted into LPE and LPC, respectively [13,33]. In this study, CCTα knockout and overexpression were found to affect PC synthesis through the CDP–choline pathway, thus influencing the synthesis of lipids in other pathways; the balance between phospholipid compounds has been suggested to further affect the formation of LDs [34]. Our findings strongly suggest that CCTα affects LD formation through PC synthesis in BMECs.

The coalescence process of LDs, called LD fusion, is a dynamic mode of LD growth [35]. The biosynthesis of large LDs is attributable to a greater accumulation of TG in cells [36], or to a greater susceptibility to LD fusion [37]. In the present study, after the knockout of the CCTα gene, small LDs decreased, and large LDs increased (Figure 3), thereby suggesting the occurrence of LD fusion. Subsequently, we observed that the frequency of LD fusion was significantly higher in CCT-KO cells than in the control and CCT-OE cells, as observed with a live cell workstation (Figure 6). Thus, the knockout of CCTα had a significant effect on promoting LD fusion in mammary epithelial cells, which increased the LD size.

LDs are energy storage organelles whose metabolic pathways are closely associated with cell homeostasis. LD fusion, lipid phagocytosis, and lipolysis are important factors regulating the LD size [38]. Lipophagy, the autophagic degradation of intracellular LDs, is involved in the regulation of intracellular lipid storage, intracellular free lipid levels, and energy balance [39]. Phospholipids have been found to be associated with autophagy, and autophagy membranes are rich in PC [40].

In this study, in CCT-OE cells, the expression of the key autophagy protein LC3 and the number of autophagic lysosomes were elevated, thus indicating autophagy activation. Andrejeva has found that increased choline phospholipid production and the activation of PCYT1A (the rate-limiting enzyme of phosphatidylcholine synthesis CCTα) may lead to the development of autophagy [41]. Similarly, in this study, CCTα overexpression and increased PC synthesis might have resulted in autophagy activation. After the addition of autophagy inhibitors, the number of LDs and the proportion of small LDs in the CCT-OE group decreased (Figure 7H). Further observations through transmission electron microscopy revealed that autophagy vesicles targeted LDs for degradation (Figure 7I), thereby indicating the occurrence of lipophagy. LD biogenesis and microautophagy have been found to be regulated by PC biosynthesis in yeast [42]. Similarly, we observed that LD formation and lipophagy are regulated by PC biosynthesis in mammary epithelial cells.

## 4. Materials and Methods

### 4.1. Ethics Approval

The study was conducted on farms in Henan Province. All animal studies were conducted in accordance with the experimental practices and standards approved by the Animal Welfare and Research Ethics Committee at Henan Agricultural University.

### 4.2. Bovine Mammary Gland Tissue

Percutaneous biopsies of the right or left rear quarter of the mammary gland in eight multiparous Holstein dairy cows (150 ± 15 DIM, 570 ± 32.5 kg weight, 32.0 ± 3.5 kg milk/d) were collected at −30, +1, +15, and +30 d relative to parturition, according to previous procedures [43].

### 4.3. Cell Culture

BMEC culture was conducted as previously described [17] in DMEM containing 10% fetal bovine serum and 1% penicillin and streptomycin (HyClone, Logan, UT, USA), in a 5% CO_2_ incubator at 37 °C. An LD model was established by the addition of 100 μmol/L LA to well-grown cells (in DMEM containing 100 μmol/L LA (Thermo Scientific, Waltham, MA, USA), 1 mg/mL bovine serum albumin, 5 μg/mL insulin, 1 μg/mL prolactin, 1 μg/mL of serum albumin, and 0.5 μg/mL hydrocortisone) for subsequent experiments.

### 4.4. Oil Red O Staining

Cells were washed three times with phosphate buffer solution (PBS) and fixed with 4% paraformaldehyde for 20 min at room temperature. After three washes in PBS, cells were washed with isopropanol and stained with Oil Red O working solution (G1260, Solarbio, Beijing, China) for 25 min. The cells were then washed with PBS, and the nuclei were restained with hematoxylin for 1–2 min. The cell was removed and placed on the slide. Each group underwent three repetitions. After slide sealing, the LDs were photographed and observed under a microscope. Cell Sens standard software (version 1.13; Olympus, Center Valley, PA, USA) was used to measure LDs diameter. ImageJ version 1.54p was used to determine the statistical number of LDs.

### 4.5. Immunofluorescence

Cells were treated with LA (100 μmol/L) for 0 h, 0.5 h, 1 h, 3 h, 6 h, 12 h, or 24 h, then rinsed with PBS three times; fixation was conducted with 4% paraformaldehyde for 20 min. Subsequently, cells were rinsed with PBS and permeated with 0.1% Triton X-100 for 30 min. After being rinsed with PBS, cells were blocked with 10% FBS for 1 h at room temperature, incubated with primary antibody to CCTα (6931, Cell Signaling, Danvers, MA, USA) for 1 h at room temperature, and Alexa Fluor TM 488-conjugated sheep anti-rabbit IgG (H+L) (A11034, Thermo Fisher Scientific, Waltham, MA, USA) for 1 h at room temperature. LDs were stained with Nile red for 15 min. The DAPI staining of nuclei was performed for 10 min. The cells were taken out and placed on slides, then sealed with an anti-fluorescence quenching agent. After drying, the slides were visualized with an Olympus IX73 fluorescence microscope equipped with an Olympus DP80 digital camera.

### 4.6. Knockout and Overexpression of CCTα

The CCTα knockout cell lines (CCT-KO) and overexpression cell lines (CCT-OE) were constructed as previously reported [15]. Briefly, CCTα was disrupted in cells through CRISPR/Cas9 gene editing with a guide RNA designed (http://crispor.tefor.net) to target exon 3 (5′-CTCCTAAGTAAGACTTTCCTCCAA-3′) of CCTα. The guide RNA was cloned in a plasmid (Supplementary Figure S1) and transfected into BMECs. Cells were screened with puromycin, and individual cell clones were isolated through limiting dilution. Genomic DNA was isolated from individual clones, and PCR was performed with CCTα-Test primer (Supplementary Figure S2). PCR products were purified and subjected to sequencing (Supplementary Figure S3). Overexpression plasmids were generated by cloning of the amplified CCTα sequence (Supplementary Figure S4) into the TSC vector. Cells were transfected and screened with puromycin, and overexpression cell lines were isolated through limiting dilution. The overexpressed gene was sequenced (Supplementary Figure S5). CCTα mRNA and protein expression were measured with qPCR and Western blotting, respectively.

### 4.7. Western Blotting

BMECs were washed and scraped into cold PBS, then resuspended in RIPA lysis buffer with protease inhibitors. The protein concentrations of cell lysates were determined with a BCA assay kit. Protein samples (10 μg) were separated by SDS-PAGE and transferred to a PVDF membrane. The membranes were then incubated in blocking solution for 2 h at room temperature. After the membranes were washed, they were hybridized overnight at 4 °C with rabbit anti-LC3B (ab48394, Abcam, Cambridge, UK) and rabbit anti-p62 (ab101266, Abcam). After the membranes were washed with TBST buffer for 5 min, they were incubated with goat anti-rabbit antibodies (Protein Technology, Tucson, AZ, USA) for 1 h at room temperature. The membranes were washed with TBST buffer and detected with an ECL kit (Thermo Scientific, Waltham, MA, USA) and Amersham Imager 600 (GE, Boston, MA, USA). The intensities of immunoreactive protein bands were quantified in ImageJ version 1.54p and normalized to β-actin.

### 4.8. Real-Time Fluorescence Quantification RCR(RT-qPCR)

Briefly, total RNA was isolated from bovine mammary gland tissues and BMECs with TRIzol, according to the manufacturer’s instructions. First-strand cDNA was synthesized with a Prime Script RT Reagent kit with gDNA Eraser (Takara, Osaka, Japan), and quantitative real-time PCR analysis was performed in a 7500 fast Real-Time PCR System (ABI, Los Angeles, CA, USA). The qPCR primer sequences for the CCTα gene were F: CTATGTGGATGAGGTGGTG and R: TCGCTCCTGTAAGTGGTATT. The data were analyzed with the 2^−ΔΔCt^ method, and the relative target gene expression was normalized to that of glyceraldehyde-3-phosphate dehydrogenase (GAPDH).

### 4.9. Living Cell Observation

Cells treated with LA were stained with BODIPY493/503 (10 μg/mL) for 30 min and washed with PBS; DMEM containing LA was then added. The culture plates were placed in a Nikon Live cell workstation and incubated at 37 °C under 5% CO_2_ for viable cell observation. The light field and fluorescence channel were set up for observation under a 60× magnification lens, and photographs were taken every 5 min for a total of 60 min.

### 4.10. Thin Layer Chromatography

Cells were collected, and 4 mL of extract (methanol–chloroform = 2:1) was added. After vortexing, ultrasonication was performed. After centrifugation, the chloroform layer was collected, 1% sodium chloride solution was added, and the mixture was vortexed. After standing, the chloroform layer was collected. After nitrogen drying, methanol was added for re-dissolution. The PC, PE standards, and previously extracted samples were sequentially spotted on activated GF254 silica gel plates. The plates were placed in an unfolding agent (chloroform–methanol–water = 65:25:4) for 15–20 min and heated with 5% phosphomolybdic acid (ethanol preparation) for color development.

### 4.11. Transmission Electron Microscopy (TEM)

After 24 h of LA treatment, cells were collected, the medium was discarded, 1 mL fresh medium was added, and cells were incubated for 5 min. Cells were scraped once, collected in 2 mL round bottom centrifugal tubes, and prefixed by the addition of 100 μL 2.5% glutaraldehyde fixative. After centrifugation at 500× *g* for 5 min, the cells were fixed with 2.5% glutaraldehyde for 4 h. Cells were rinsed four times with washing buffer, fixed in 1% osmic for 2 h at room temperature, and rinsed four times in PBS. Samples were then dehydrated in a graded ethanol series, infiltrated in epoxy 812 overnight, polymerized (37 °C, 12 h; 45 °C, 12 h; 60 °C, 24 h), trimmed, and used to make ultra-thin slices. Saturated uranyl acetate staining was performed for 20 min, and was followed by washing, drying, and staining with lead citrate solution for 5 min. Cells were subsequently washed, dried, and viewed under a Jeol 7800 transmission electron microscope (Tokyo, Japan).

### 4.12. Co-Localization of Lysosomes and LDs

A total of 1 × 10^3^ cells were seeded into a 20 mm glass bottom dish, and 100 μmol/L LA was added when the cells reached 75% confluence after 24 h. Cells were incubated for another 24 h. The medium was discarded, and cells were washed once with PBS. BODIPY493/503 (10 μg/mL) staining solution was added, and cells were incubated in the dark for 30 min. The staining solution was discarded, and cells were washed three times with PBS. Subsequently, 1 nmol/L Lyso-tracker solution was added, and cells were placed in an incubator in the dark for 15 min. The staining solution was discarded, cells were washed three times with PBS, and normal medium was added. Finally, the cells were observed under a laser confocal microscope and photographed.

### 4.13. Statistical Analysis

Statistical analyses of all data were performed in SPSS 20.0 (IBM, Armonk, NY, USA). Prism 9 (GraphPad, La Jolla, CA, USA) was used for mapping analysis. All data were repeated at least three times, and the mean value was calculated. The *t*-test was used for comparison between two groups, and one-way analysis of variance was used for data analysis of three or more groups. The real-time fluorescence quantification PCR results were analyzed with the 2^−ΔΔCt^ method, with GAPDH as a reference gene. Each experiment was performed at least in triplicate. * *p* < 0.05 indicated a significant difference.

## 5. Conclusions

In summary, our findings define a role of CCTα in regulating LD formation in BMECs. The formation of LDs is determined by the LD size and number, which in turn are affected by the synthesis of phospholipids. CCTα further modulates the formation of LDs by regulating their fusion and lipophagy in mammary epithelial cells. These results provide new insights into CCTα as a target gene affecting the nutritional function of milk fat globules in dairy cows.

## Figures and Tables

**Table 1:** Effect of CCTα on cell lipid quantity.

Group Name	All Significance Difference Lipids	Down	Up
CCT-KO vs. CCT-OE	688	594	74
NC vs. CCT-KO	815	48	767
NC vs. CCT-OE	190	50	140

## Data Availability

The original contributions presented in this study are included in the article/Supplementary Materials. Further inquiries can be directed to the corresponding author(s).

## Supplementary Materials

The supporting information can be downloaded at: https://www.mdpi.com/article/10.3390/ijms26052135/s1.
