# Supplementary material for: Phosphatidylcholine Cytidine Transferase α (CCTα) Affects LD Formation Through Fusion and Lipophagy in Bovine Mammary Epithelial Cells
Source: Int J Mol Sci. 2025 Feb 27;26(5):2135. doi: 10.3390/ijms26052135 (PMC11901133; doi:10.3390/ijms26052135)
Supplement: Supplementary file 1 [file ijms-26-02135-s001.zip › ijms-3424399-supplementary.pdf]

Supplementary Materials:

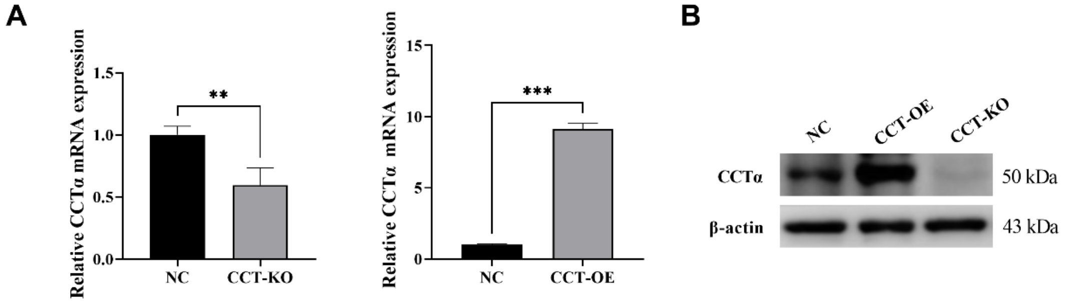

Supplementary **Figure S1.** Construction of CCTα knockout and overexpression cell lines. (A) mRNA expression of the CCTα gene. (B) Protein expression of CCTα. All data are presented as mean ± SD; **P < 0.01, ***P < 0.001.

CCT gene ID NCBI: 100125779

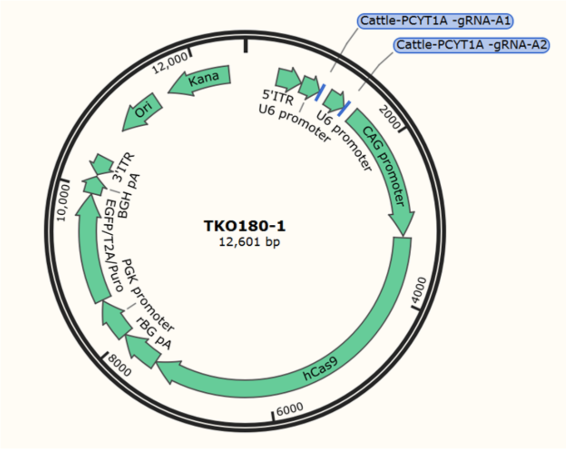

Supplementary Figure S2. CCT-KO Plasmid Map

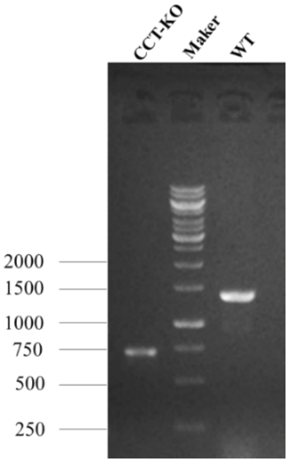

Supplementary Figure S3. PCR Identification

ATCTGGTAGAGGAGCTGTCATTCTGGACCCAGTTG -del 730 bp-in 37 bp-
TCAGACTGTCACATTTTTAATAGTCTTCAGAATTT

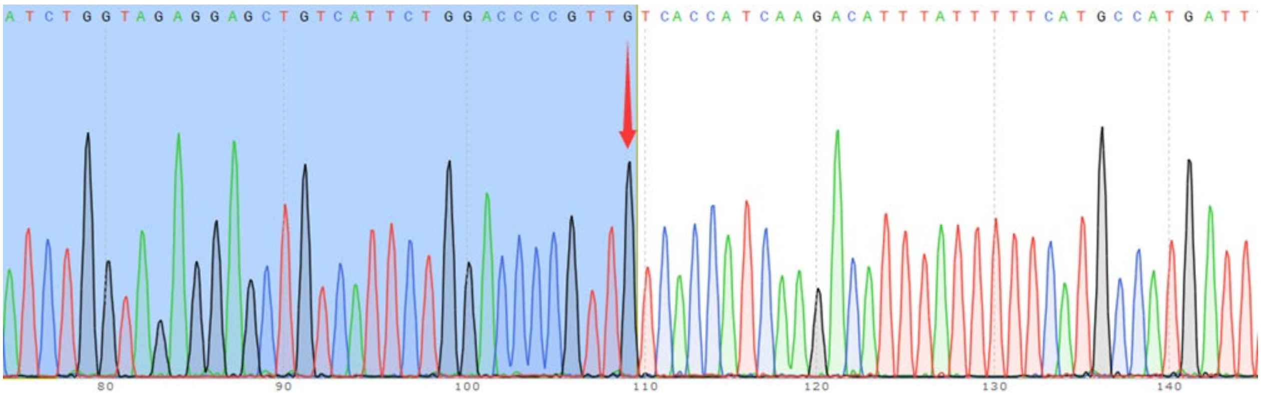

Supplementary Figure S4. CCT-KO sequencing

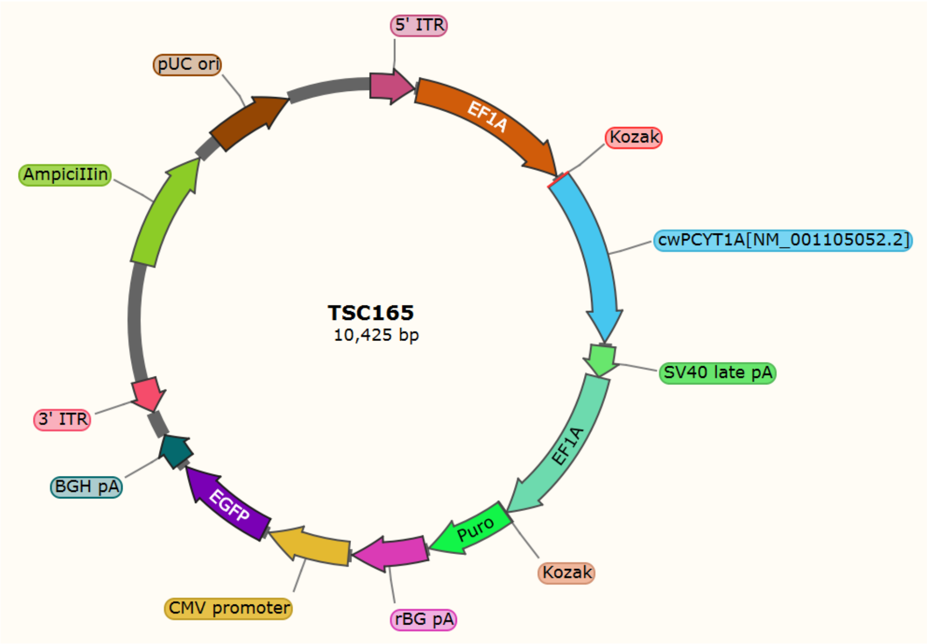

Supplementary Figure S5. CCT-OE Plasmid Map

```
5'  caaaaaagcaggctgccaccatggatacacagaattcagccaaagtcaacacaaggaagaggaggaaagagacacctgga
                                                                                      80
3'  gtttttttcgtccgacggtggtacctatgtgtcttaagtcggtttcagttgtgttccttctcctcctttctctgtggacct

    cccaatggggcaacagaagaagatggaattccttcaaaaatgccacgctgtgcaattggtttacggcagccagcgccttt
                                                                                      160
    gggttaccccgttgtcttcttctaccttaaggaagtttttacggtgcgacacgttaaccaaatgccgtcggtcgcggaaa

    ttctgatgaaattgaagtagactttagtaagccctatgtcagggtaactatggaagaagccagcagaggaactccttgtg
                                                                                      240
    aagactactttaacttcatctgaaatcattcgggatacagtcccattgataccttcttcggtcgtctccttgaggaacac

    aacggcctgtgagagtttatgcagatggaatatttgacttatttcattctggtcatgcccgggctctgatgcaagcaaag
                                                                                      320
    ttgccggacactctcaaatacgtctaccttataaactgaataaagtaagaccagtacgggcccgagactacgttcgtttc

    aacctttttccctaatacatacctcattgtgggagtttgcagtgatgagctaacacacaacttcaaaggcttcaccgtgat
                                                                                      400
    ttggaaaaagggattatgtatggagtaacaccctcaaacgtcactactcgattgtgtgttgaagtttccgaagtggcacta

    gaatgaaaatgagcgctatgatgcagtgcagcactgccgctatgtggatgaggtggtgaggaatgccccctggaccctga
                                                                                      480
    cttactttactcgcgatactacgtcacgtcgtgacggcgatacacctactccaccactccttacggggggacctgggact

    caccagagttcctggcagaacaccggattgattttgtggctcatgatgacatcccttattcttctgctgggagtgatgat
                                                                                      560
    gtggtctcaaggaccgtcttgtggcctaactaaaacaccgagtactactgtagggaataagaagacgaccctcactacta

    gtttataagcacatcaaggaagcaggcatgtttgctccaacacagaggacagaaggtatctccacatcagacatcatcac
                                                                                      640
    caaatattcgtgtagttccttcgtccgtacaaacgaggttgtgtctcctgtcttccatagaggtgtagtctgtagtagtg

    tcgaattgtccgtgactatgatgtgtatgccaggaggaacctccagaggggctacacagcaaaggaactcaatgtcagct
                                                                                      720
    agcttaacaggcactgatactacacatacggtcctccttggaggtctccccgatgtgtcgtttccttgagttacagtcga

    ttatcaatgagaagaaataccacttacaggagcgagttgacaaagtaaagaagaaagtgaaagatgtggaggaaaagtca
                                                                                      800
    aatagttactcttctttatggtgaatgtcctcgctcaactgtttcatttcttctttcactttctacacctccttttcagt

    aaggaatttgttcagaaggtggaagaaaaaagcattgacctcattcagaaatgggaagagaaatcccgagaattcattgg
                                                                                      880
    ttccttaaacaagtcttccaccttcttttttcgtaactggagtaagtctttacccttctctttagggctcttaagtaacc

    aagtttcctggaaatgtttggtccagaaggagcgctgaaacatatgctgaaagaggggaaaggccggatgctgcaggcca
                                                                                      960
    ttcaaaggacctttacaaaccaggtcttcctcgcgactttgtatacgactttctcccctttccggcctacgacgtccggt

    tcagtccaaggcagagtcccagcaacagccccactcgtgaacgttcccccctctccctctttccggtggcccttctctggc
                                                                                      1040
    agtcaggttccgtctcagggtcgttgtcggggtgagcacttgcaaggggggagagggagaaaggccaccgggaagagaccg

    aagacttccccgccttcctccccaacaaacctctccagacacaagtctgcagcctatgatatcaggtatagttacctgga
                                                                                      1120
    ttctgaaggggcggaaggaggggttgtttggagaggtctgtgttcagacgtcggatactatagtccatatcaatggacct

    acagaacaaatttggctcctatgttctgctcgcccaggtgcagcctggcttgacgaaaaatgatgcctatggtccacaga
                                                                                      1200
    tgtcttgtttaaaccgaggatacaagacgagcgggtccacgtcggaccgaactgctttttactacggataccaggtgtct

    gccgacagggtggacacaccgaggaaagtgttgatccatagacccagctttcttgtacaaagt      3'
                                                                            1263
    cggctgtcccacctgtgtggctcctttcacaactaggtatctgggtcgaaagaacatgtttca      5'
```

Supplementary Figure S6. CCT-OE sequencing

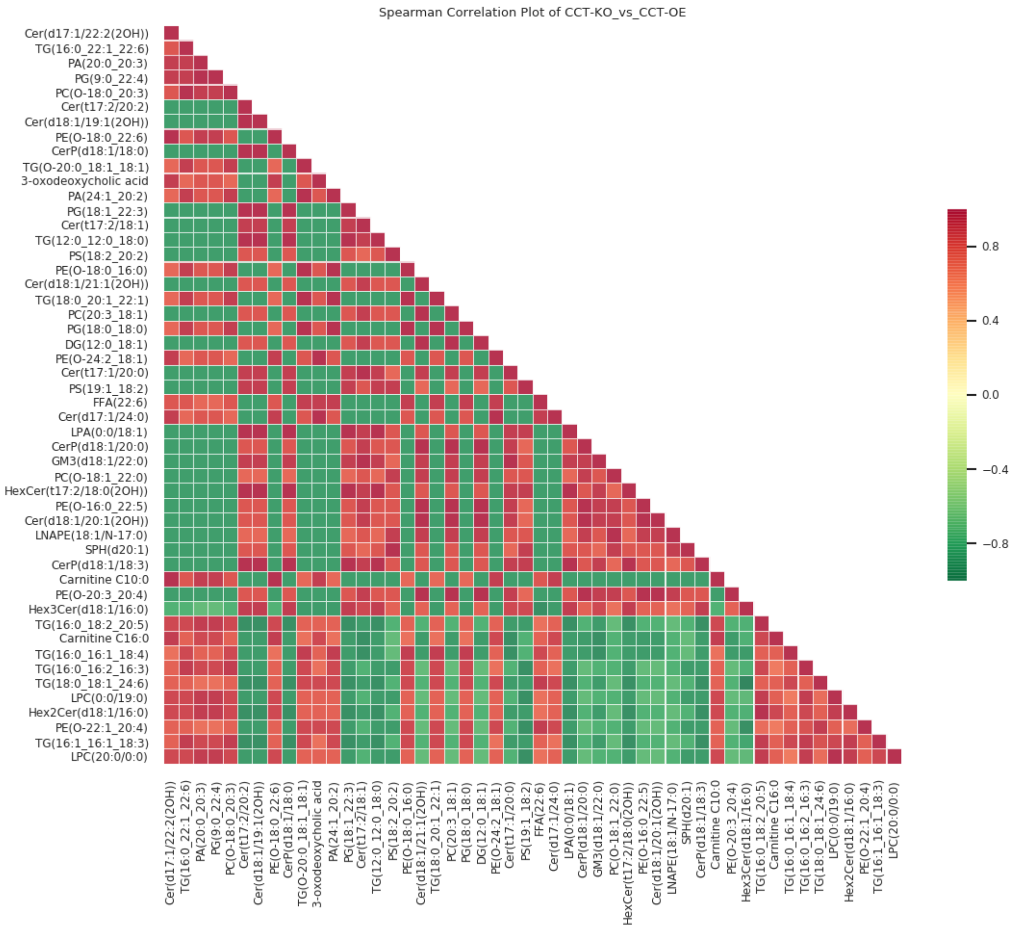

Supplementary Figure S7. CCT-KO and CCT-OE Differential lipid correlation sharing

Top 50 lipid correlation coefficient. The top 50 differential lipids with the largest VIP value are selected. Red represents a strong positive correlation, green represents a strong negative corre-lation, and darker color indicates a greater absolute value of the correlation coefficient between samples.
